# Phase I study of IMGN901, a CD56-targeting antibody-drug conjugate, in patients with CD56-positive solid tumors

**DOI:** 10.1007/s10637-016-0336-9

**Published:** 2016-03-09

**Authors:** Manisha H. Shah, Paul Lorigan, Mary E. R. O’Brien, Frank V. Fossella, Kathleen N. Moore, Shailender Bhatia, Maurice Kirby, Penella J. Woll

**Affiliations:** Ohio State University College of Medicine, Columbus, OH 43210 USA; University of Manchester/Christie NHS Foundation Trust, Manchester, M20 4BX UK; Royal Marsden Hospital, Sutton, Surrey SM2 5PT UK; Department of Thoracic/Head and Neck Medical Oncology, Division of Cancer Medicine, The University of Texas MD Anderson Cancer Center, Houston, TX 77030 USA; University of Oklahoma Health Sciences Center, Oklahoma City, OK 73104 USA; Department of Medicine, University of Washington School of Medicine and Fred Hutchinson Cancer Research Center, Seattle, WA 98109 USA; ImmunoGen, Inc., Waltham, MA 02451 USA; Academic Unit of Clinical Oncology, Weston Park Hospital, University of Sheffield, Whitham Road, Sheffield, S10 2SJ UK

**Keywords:** Antibody-drug conjugate, CD56, IMGN901, DM1, Lorvotuzumab mertansine

## Abstract

*Background* IMGN901 is a CD56-targeting antibody-drug conjugate designed for tumor-selective delivery of the cytotoxic maytansinoid DM1. This phase 1 study investigated the safety, tolerability, pharmacokinetics, and preliminary activity of IMGN901 in patients with CD56-expressing solid tumors. *Methods* Patients were enrolled in cohorts of escalating IMGN901 doses, administered intravenously, on 3 consecutive days every 21 days. A dose-expansion phase accrued patients with small cell lung cancer (SCLC), Merkel cell carcinoma (MCC), or ovarian cancer. *Results* Fifty-two patients were treated at doses escalating from 4 to 94 mg/m^2^/day. The maximum tolerated dose (MTD) was determined to be 75 mg/m^2^. Dose-limiting toxicities included fatigue, neuropathy, headache or meningitis-like symptoms, chest pain, dyspnea, and myalgias. In the dose-expansion phase (*n* = 45), seven patients received 75 mg/m^2^ and 38 received 60 mg/m^2^ for up to 21 cycles. The recommended phase 2 dose (RP2D) was established at 60 mg/m^2^ during dose expansion. Overall, treatment-emergent adverse events (TEAEs) were experienced by 96.9 % of all patients, the majority of which were Grade 1 or 2. The most commonly reported Grade 3 or 4 TEAEs were hyponatremia and dyspnea (each 8.2 %). Responses included 1 complete response (CR), 1 clinical CR, and 1 unconfirmed partial response (PR) in MCC; and 1 unconfirmed PR in SCLC. Stable disease was seen for 25 % of all evaluable patients who received doses ≥60 mg/m^2^. *Conclusions* The RP2D for IMGN901 of 60 mg/m^2^ administered for 3 consecutive days every 3 weeks was associated with an acceptable tolerability profile. Objective responses were observed in patients with advanced CD56+ cancers.

## Introduction

The glycoprotein CD56, also known as NCAM1, is a member of the neural cell adhesion molecule family [1] that plays important functional roles during development, nervous system differentiation, and immune surveillance [2]. Primarily expressed in neuroendocrine, natural killer, and T cell lineages [3], aberrant CD56 expression is seen in a variety of hematological malignancies (e.g. multiple myeloma, myelocytic and lymphocytic leukemia) as well as solid tumors (most notably small cell lung cancer [SCLC], Merkel cell carcinoma [MCC], and ovarian cancer) [4–8]. Indeed, for solid tumors, CD56 serves as a diagnostic biomarker to identify those of neuroendocrine origin, including MCC and SCLC [9, 10]. Effective treatment options for these two tumor types are limited, particularly within the context of advanced and/or refractory disease [11, 12]. CD56 has thus emerged as an attractive molecular candidate for the design of novel, targeted therapeutic strategies for improving patient outcomes in these indications.

IMGN901 (lorvotuzumab mertansine) is an antibody-drug conjugate (ADC), consisting of a humanized anti-CD56 antibody to which the tubulin-binding maytansinoid DM1 is covalently conjugated via a stable disulfide linker [13]. IMGN901 targets CD56 at the cell surface and, upon antigen binding, becomes internalized - resulting in the intracellular release of DM1 [14], which in turn promotes disruption of microtubule assembly, G2/metaphase arrest, and ultimately apoptosis [15–17]. In preclinical models, IMGN901 has shown high-affinity, antigen-specific binding and robust antitumor activity in CD56-positive (CD56+) tumors [18].

A first-in-human, open label phase I/II clinical trial has previously been conducted in patients with relapsed or refractory CD56+ SCLC and other CD56+ solid tumors where IMGN901 was administered weekly for four consecutive weeks on a six-week cycle [NCT00065429]. The results of that dose-escalation study revealed that IMGN901 displayed favorable pharmacokinetics, a manageable safety profile (including no cardiac, thyroid or adrenocortical toxicities despite CD56 expression in those tissues), and encouraging signs of activity [19, 20]. These findings prompted further evaluation of IMGN901 in the same target populations using an alternative dosing schedule. Here we report on an open label, Phase I trial of IMGN901 dosed on days 1–3 of a 21-day cycle in patients with CD56+ tumors. This regimen was selected based on prior human data showing a relatively short half-life for low doses of IMGN901. This study was undertaken to determine the tolerability, safety, and maximum tolerated dose (MTD) of IMGN901 on this schedule. Secondary assessments included pharmacokinetic profiling and preliminary assessment of clinical activity.

## Patients and methods

This was a single-agent, multicenter, open-label, non-randomized, Phase I study with expansion at the MTD that involved 9 sites in the United States and the United Kingdom. The study was conducted in accordance with the US Food and Drug Administration regulations, the International Conference on Harmonisation Guidelines for Good Clinical Practice and the Declaration of Helsinki. The study was compliant with Institutional Review Board and Independent Ethics Committee requirements. All patients provided written informed consent in accordance with federal, local and institutional guidelines. This trial was registered at Clinical trials.gov (NCT00346385).

### Eligibility criteria

During dose escalation, enrollment was open to patients who had histologically or cytologically proven, measurable, relapsed or refractory SCLC, neuroendocrine pulmonary tumors, metastatic MCC or carcinoid tumors, or other CD56+ solid tumors. Tumor CD56 expression was assessed prior to enrollment or at the earliest opportunity. With the exception of carcinoid and neuroendocrine tumors (which may not have been treated with chemotherapy previously), patients who had received one to three prior therapies were eligible. In the MTD expansion phase, enrollment was restricted to patients with relapsed or refractory SCLC, locally advanced or metastatic MCC, or ovarian cancer. Patients with SCLC who had received one prior chemotherapy regimen were eligible, whereas patients with MCC or ovarian cancer may have received more than one prior chemotherapy regimen. Patients with resistant or refractory ovarian cancer must have received at least one platinum-based regimen.

Eligible patients were required to be ≥18 years of age; have an Eastern Cooperative Oncology Group (ECOG) performance status ≤2; and a life expectancy of at least 3 months. Adequate hematology laboratory values and organ function were required. Patients were excluded if they had known hypersensitivity to monoclonal antibody therapy; brain metastases; or a previous malignancy with <3-year disease-free interval other than basal cell carcinoma or carcinoma in situ of the cervix.

### Study design and drug administration

Enrollment of a maximum of 100 patients was planned. In the dose-escalation phase, patients received IMGN901 intravenously (IV) at 4, 8, 16, 24, 36, 48, 60, 75, or 94 mg/m^2^ for a maximum of 4 cycles or until intolerable toxicity, patient withdrawal or progressive disease (PD). Originally, IMGN901 was to be infused over 1 h; later, the infusion rate was reduced to 40 min. However, because of the onset of Grade 2 or higher headaches in individuals while being infused at higher doses, the protocol was amended to establish an infusion rate of 1 mg/min and prophylactic measures for all patients. These included 8 mg of oral dexamethasone (or equivalent) twice a day on the day prior to the infusion; 10 mg of IV dexamethasone 30 min prior to the infusion; and acetaminophen (500–650 mg). Patients experiencing objective responses as per RECIST 1.0 criteria [21], or those with stable disease (SD) showing clinical benefit were eligible to receive additional cycles at the same dose as that of their last treatment cycle until PD or intolerability. In the expansion phase, IMGN901 administration began at the MTD, defined as the highest dose at which <2 of six patients experienced a dose limiting toxicity (DLT), defined as grade 4 neutropenia ≥5 days, grade 4 thrombocytopenia, neutropenic infection, or any grade 3 or 4 non-hematologic toxicity (except nausea, vomiting, diarrhea, and alopecia).

### Safety evaluation

All patients who enrolled and received at least one dose of study drug underwent safety evaluation (*n* = 97). AEs were assessed at each visit and graded according to the National Cancer Institute Common Terminology Criteria for Adverse Events Version 4.0.

### Efficacy evaluation

Preliminary assessment of efficacy was performed using the RECIST response criteria version 1.0. A CT scan or MRI was performed at screening, at the end of treatment Cycles 2 and 4, at Day 22/early study treatment termination visit, at the 1-month follow-up visit, and at each short-term follow-up visit (every 6 weeks after the 1-month follow-up visit until PD or start of alternate anti-neoplastic therapy). Efficacy parameters included objective response rate (ORR), duration of response (DOR), time to progression (TTP), progression free survival (PFS) and overall survival (OS) for up to 3 years. Patients were evaluable for efficacy if they had a post-baseline radiologic or clinical assessment.

### Phamacokinetics evaluation

Blood samples for pharmacokinetic (PK) characterization of intact conjugate, total huN901 antibody, human anti-human antibody, and human anti-drug antibody were collected from all patients at pre-specified time points. PK summary measures were calculated for each patient. PK analysis was performed using the standard algorithms of the non-compartmental PK analysis program (201), WinNonlin, Professional version 6.1.0.173 (Pharsight Corporation, Mountain View, California).

### Statistical methods

Descriptive statistics for continuous variables were summarized using sample size (n), mean, median, standard deviation, minimum, and/or maximum. Patients who discontinued IMGN901 and started alternate anti-neoplastic therapy before documentation of PD were censored at the time of their last visit prior to initiation of alternate anti-neoplastic therapy. Patients who died or were lost to follow-up before documentation of PD or who did not progress before data cut-off were censored at the time of their last visit.

Any event with the same onset date as start of study treatment or later was reported as treatment emergent. Time-to-event variables (DOR, TTP, PFS, and OS) were based on Kaplan-Meier estimations. Baseline was defined as the last available assessment prior to Day 1, Cycle 1.

All response-evaluable patients who had a post-baseline assessment (clinical or radiologic per RECIST 1.0) were included in the Clinical Benefit Rate (CBR) analysis (complete response [CR], partial response [PR] or SD of ≥75 days in duration). A subset of this evaluable population, excluding those without post-baseline radiologic assessments, was used to evaluate the Disease Control Rate (DCR; CR, PR, or SD ≥75 days by radiologic assessment).

## Results

### Patient characteristics

The study accrued 97 patients, 52 in the dose-escalation phase and 45 in the expansion phase. In the dose-escalation phase, at least four patients were treated at each of the first 8 dose levels, ranging from 4 mg/m^2^ to 75 mg/m^2^, and two patients were treated at the highest dose, 94 mg/m^2^. In the expansion phase, the first seven patients were treated at a dose of 75 mg/m^2^ and the remaining 38 at 60 mg/m^2^.

The median age of the patients enrolled in the study was 58 years (range, 18–88) and the distribution of primary tumor types was SCLC (35.1 %), MCC (23.7 %), neuroendocrine carcinoma (15.5 %), and ovarian cancer (12.4 %) (Table 1). The majority of patients treated at 60 mg/m^2^ and 75 mg/m^2^ completed the study (86.4 % and 90.9 %, respectively), for an overall study completion rate of 86.6 %. Overall exposure ranged from 1 to 21 cycles of IMGN901 with total cumulative doses ranging from 22.5 mg to 6680 mg. Twenty-eight percent of the patients completed 3 or more cycles, with 10.3 % completing at least 6 cycles of study treatment. Thirteen patients did not complete Day 22 of cycle 1 as follows: eight patients withdrew due to disease progression and five withdrew due to an AE.

**Table 1:** Patient demographics and baseline characteristics

Characteristic	Dose level (mg/m^2^)				
	4–48^a^ (*n* = 29)	60 (*n* = 44)	75 (*n* = 22)	94 (*n* = 2)	Total (*n* = 97)
Median age (range), years	54.8 (18–77)	60.5 (30–78)	55.0 (29–88)	57.5 (50–65)	58 (18–88)
Gender, n (%)					
Male	20 (69.0)	13 (29.5)	7 (31.8)	0	40 (41.2)
Female	9 (31.0)	31 (70.5)	15 (68.2)	2 (100)	57 (58.8)
CD56+ tumor type, n (%)					
Carcinoid tumor	1 (3.4)	0	1 (4.5)	0	2 (2.1)
Ewing’s sarcoma	1 (3.4)	0	0	0	1 (1.0)
Lung cancer (undefined)	2 (6.9)	0	0	0	2 (2.1)
Merkel cell carcinoma	2 (6.9)	15 (34.1)	6 (27.3)	0	23 (23.7)
Neoplasm^b^	1 (3.4)	1 (2.3)	1 (4.5)	0	3 (3.1)
Neuroendocrine carcinoma	7 (24.1)	3 (6.8)	4 (18.2)	1 (50.0)	15 (15.5)
Non-small cell lung cancer	2 (6.9)	0	0	0	2 (2.1)
Ovarian cancer	0	12 (27.3)	0	0	12 (12.4)
Sarcoma	0	0	1 (4.5)	0	1 (1.0)
Small cell lung cancer	12 (41.4)	13 (29.5)	8 (36.4)	1 (50.0)	34 (35.1)
Thyroid cancer	1 (3.4)	0	0	0	1 (1.0)
Vulvar cancer	0	0	1 (4.5)	0	1 (1.0)
Previous treatment, n (%)					
Any	29 (100)	44 (100)	22 (100)	2 (100)	97 (100)
Chemotherapy	29 (100)	44 (100)	22 (100)	2 (100)	96 (99.0)
Radiotherapy	20 (69.0)	27 (61.4)	13 (59.1)	1 (50.0)	61 (62.9)
Surgery	13 (44.8)	25 (56.8)	13 (59.1)	2 (100)	53 (54.6)
Other^c^	2 (6.9)	4 (9.1)	1 (4.5)	0	7 (7.2)
Patient disposition					
Completed Cycle 1	26 (89.7)	38 (86.4)	20 (90.9)	0	84 (86.6)
Did not complete Cycle 1					
*-Disease deterioration*	3 (10.3)	4 (9.1)	1 (4.5)	0	8 (8.2)
*-AE*/*unacceptable toxicity*	0	2 (4.5)	1 (4.5)	2 (100)	5 (5.2)

^a^Combined data; 4 patients received 4 mg/m^2^, 4 received 8 mg/m^2^, 6 received 16 mg/m^2^, 4 received 24 mg/m^2^, 4 received 36 mg/m^2^, and 7 received 48 mg/m^2^

^b^Neoplasm included a right hallus tumor, an unknown primary tumor, and a tumor of the right foot

^c^Other included hormonal, immunologic or biologic therapies

### Safety and tolerability

Grade 4 fatigue in cycle 1 and Grade 2 peripheral neuropathy that progressed to Grade 3 in cycle 2 were reported as DLTs in the 16 mg/m^2^ (*n* = 1) and 48 mg/m^2^ (*n* = 1) cohorts. At the 75 mg/m^2^/day dose level, an initial cohort of four patients was enrolled. One patient experienced a DLT of Grade 4 headache associated with meningitis-like symptoms, prompting a reduction in the infusion rate. An additional four patients were treated with the slowed infusion rate, two of whom developed Grade 2 headaches that in turn prompted the administration of prophylactic steroids. Of a total of seven additional patients in the steroid pre-treated 75 mg/m^2^ cohort, one developed neuropathy in cycle 1 that worsened to a DLT (Grade 3 paresthesia) in cycle 3. Dose escalation continued to 94 mg/m^2^ with two patients who experienced pain-related DLTs on day 8 of cycle 1 (Grade 3 headache, pain, and chest pain in one patient and Grade 3 myalgia in the other). Therefore, the 75-mg/m^2^ dose was declared the MTD. However, because three of the first seven patients treated at the 75-mg/m^2^ dose experienced drug-related serious adverse events (SAEs) (generalized pain and increased chest pain [*n* = 1]; fatigue, syncope, Grade 2 neuropathy, and myalgia [*n* = 1]; and Grade 3 dyspnea, Grade 2 myalgia, chest pain, and fatigue [*n* = 1]), a dose of 60 mg/m^2^/day was explored, and ultimately confirmed as the recommended phase II dose (RP2D).

Ninety-four patients (96.9 %) reported a treatment-emergent adverse event (TEAE), the majority of which were Grade 1 or 2; with nausea (43.3 %), fatigue (39.2 %), and constipation (37.1 %) being the most commonly reported. Table 2 summarizes those TEAEs reported for ≥5 % of patients that were assessed as possibly, probably, or definitely related (“related”) to study treatment (75.3 %). The most common treatment-related TEAEs were nausea (30.9 %), fatigue (27.8 %), headache (26.8 %), peripheral neuropathy (17.5 %), and vomiting (16.5 %).

**Table 2:** Related treatment-emergent adverse events reported in ≥5 % of patients (all grades)

Related TEAEs, n (%)^a^	Dose level (mg/m^2^)				
	4–48^b^ (*n* = 29)	60 (*n* = 44)	75 (*n* = 22)	94 (*n* = 2)	Total (*n* = 97)
Any AE	16 (55.2)	36 (81.8)	19 (86.4)	2 (100)	73 (75.3)
Blood and lymphatic system	1 (3.4)	3 (6.8)	3 (13.6)	0	7 (7.2)
Gastrointestinal disorders	9 (31.0)	27 (61.4)	14 (63.6)	2 (100)	52 (53.6)
Constipation	2 (6.9)	4 (9.1)	2 (9.1)	0	8 (8.2)
Diarrhea	1 (3.4)	8 (18.2)	3 (13.6)	1 (50.0)	13 (13.4)
Nausea	6 (20.7)	18 (40.9)	6 (27.3)	0	30 (30.9)
Vomiting	6 (20.7)	7 (15.9)	3 (13.6)	0	16 (16.5)
General/IV site disorders	8 (27.6)	20 (45.5)	11 (50.0)	2 (100)	41 (42.3)
Chest pain	0	0	4 (18.2)	1 (50.0)	5 (5.2)
Fatigue	4 (13.8)	14 (31.8)	8 (36.4)	1 (50.0)	27 (27.8)
Pain	0	5 (11.4)	1 (4.5)	1 (50.0)	7 (7.2)
Infections/infestations	1 (3.4)	5 (11.4)	1 (4.5)	0	7 (7.2)
Investigations	1 (3.4)	19 (43.2)	12 (54.5)	1 (50.0)	33 (34.0)
ALT	0	9 (20.5)	3 (13.6)	1 (50.0)	13 (13.4)
AST	0	9 (20.5)	6 (27.3)	1 (50.0)	16 (16.5)
GGT	0	6 (13.6)	4 (18.2)	1 (50.0)	11 (11.3)
Hemoglobin decreased	0	4 (9.1)	1 (4.5)	0	5 (5.2)
Lipase increased	0	5 (11.4)	0	0	5 (5.2)
Metabolism/nutrition disorders	2 (6.9)	13 (29.5)	5 (22.7)	0	20 (20.6)
Appetite decreased	1 (3.4)	3 (6.8)	5 (22.7)	0	9 (9.3)
Hypokalemia	1 (3.4)	5 (11.4)	1 (4.5)	0	7 (7.2)
Musculoskeletal/connective tissue	1 (3.4)	14 (31.8)	11 (50.0)	1 (50.0)	27 (27.8)
Arthralgia	0	4 (9.1)	3 (13.6)	0	7 (7.2)
Back pain	0	2 (4.5)	2 (9.1)	1 (50.0)	5 (5.2)
Myalgia	1 (3.4)	5 (11.4)	5 (22.7)	1 (50.0)	12 (12.4)
Pain in extremity	0	1 (2.3)	7 (31.8)	0	8 (8.2)
Nervous system disorder	11 (37.9)	26 (59.1)	15 (68.2)	1 (50.0)	53 (54.6)
Dysgeusia	1 (3.4)	4 (9.1)	1 (4.5)	0	6 (6.2)
Headache	6 (20.7)	11 (25.0)	8 (36.4)	1 (50.0)	26 (26.8)
Lethargy	3 (10.3)	1 (2.3)	1 (4.5)	0	5 (5.2)
Paresthesia	0	2 (4.5)	4 (18.2)	0	6 (6.2)
Peripheral neuropathy	1 (3.4)	10 (22.7)	6 (27.3)	0	17 (17.5)
Peripheral sensory neuropathy	2 (6.9)	6 (13.6)	6 (27.3)	0	14 (14.4)
Psychiatric disorders	0	6 (13.6)	1 (4.5)	0	7 (7.2)
Respiratory/thoracic/mediastinal	2 (6.9)	6 (13.6)	3 (13.6)	1 (50.0)	12 (12.4)
Skin/subcutaneous tissue	3 (10.3)	6 (13.6)	0	1 (50.0)	10 (10.3)
Vascular disorders	2 (6.9)	5 (11.4)	1 (4.5)	0	8 (8.2)

^a^Treatment-emergent AEs are AEs with an onset date on or after the date of first dosing with IMGN901; treatment-related AEs are events with the maximum relationship to IMGN901 treatment as determined by the Investigator’s assessment of possibly, probably, or definitely related

^b^Combined data; 4 patients received 4 mg/m^2^, 4 received 8 mg/m^2^, 6 received 16 mg/m^2^, 4 received 24 mg/m^2^, 4 received 36 mg/m^2^, and 7 received 48 mg/m^2^

Seventy-eight patients (80.4 %) reported a Grade 3 or 4 TEAE; 37.1 % were assessed as treatment-related. Overall, gastrointestinal (25.8 %), metabolism and nutrition (19.6 %), investigation (18.6 %), and nervous system (15.5 %) disorders accounted for the highest incidence of Grade 3 or 4 AEs. The most commonly reported were hyponatremia (8.2 %), dyspnea (8.2 %), and elevated gamma glutamyltransferase (GGT; 7.2 %). Clinically significant elevations of pancreatic enzymes (serum amylase, lipase, and/or trypsinogen) were seen in 16 % of the patients treated at 60 mg/m^2^ and in one of four (25 %) patients in the 24-mg/m^2^ cohort; however, only two patients experienced an AE of pancreatitis.

SAEs were reported for 53 patients (54.6 %) and treatment-related SAEs were experienced by 15 patients (15.5 %). These included constipation (3.1 %); headache (3.1 %); pain, fatigue, pain in extremity, and chest pain (2.1 %); and abdominal pain and fever (1.0 %). One patient treated at 60 mg/m^2^ experienced reversible posterior leukoencephalopathy syndrome with cortical blindness [22]. Twelve patients experienced a SAE with an outcome of death. However, only one of these SAEs (bronchopneumonia), which occurred in a patient treated at 75-mg/m^2^, was judged to be related to study treatment.

### Efficacy

**Fig. 1:**
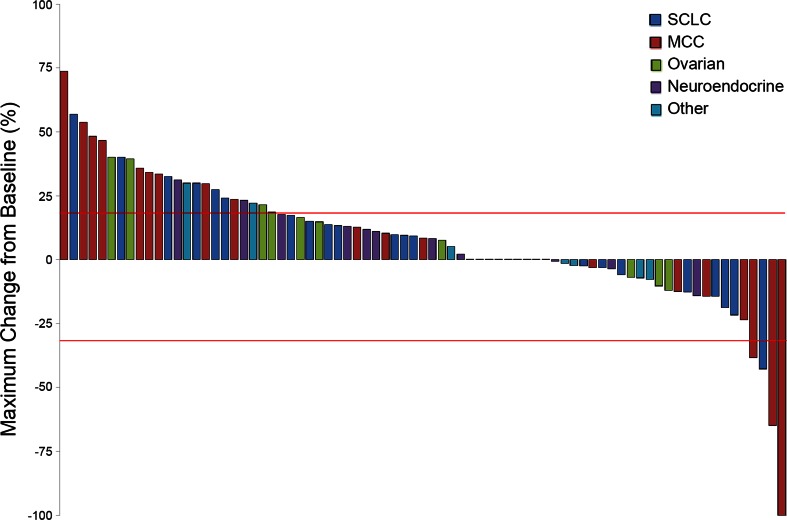
Relative change from baseline in target lesion size (at best tumor response). The maximum percent change in the sum of target lesions is displayed graphically, by histological subtype, for the 77 patients in the subset of efficacy evaluable population (*n* = 88) who had measurable disease and met RECIST criteria for evaluable post-baseline imaging measurement. SCLC, small cell lung cancer; MCC, Merkel cell carcinoma

Clinical benefit was observed in 25.5 % of all patients, with a CBR of 27.3 % in the cohort of individuals treated at the 60 mg/m^2^ dose level (Table 3). These values included SD lasting ≥75 days being seen in 25 % of patients who received a dose of 60 mg/m^2^ or higher (*n* = 68). Four patients met RECIST criteria for objective responses (Fig. 1 and Table 4) as follows: one patient with MCC who had received IMGN901 at 36 mg/m^2^ had a confirmed CR; a second MCC patient treated at 60 mg/m^2^ had a confirmed PR that progressed to clinical CR but refused further CT assessment and, therefore, could be evaluated only by clinical assessment; and two patients, one with MCC (60 mg/m^2^) and another with SCLC (75 mg/m^2^) had unconfirmed PRs. Overall disease control rates (DCR values) were 18.2 % for all evaluable patients and 22.5 % for those treated at 60 mg/m^2^.

**Table 3:** Efficacy parameters by dose level

Efficacy Parameters	Dose level (mg/m^2^)^a^				
	4–48^a^ (*n* = 27)	60 (*n* = 44)	75 (*n* = 21)	94 (*n* = 2)	Total (*n* = 94)
Clinical benefit^b^					
No. patients with clinical benefit	5	12	6	1	24
Clinical benefit rate^c^	18.5 %	27.3 %	28.6 %	50 %	25.5 %
Time to progression^d^					
No. patients who progressed/died	24	40	17	2	83
No. patients censored	3	4	4	0	11
Median TTP [95 % CI], months	1.4 [0.7,1.9]	1.3 [1.2,1.6]	2.1	2.0 (1.0,3.3]	2.1 [1.2,1.5]
Progression-free survival^e^					
No. patients who progressed/died	25	41	17	2	85
No. patients censored	2	3	4	0	9
Median PFS [95 % CI], months	1.4 [0.7,1.9]	1.3 [1.2,1.6]	2.1	2.0 [1.0,3.3]	2.1 [1.2,1.5]

^a^Combined data; 4 patients received 4 mg/m^2^, 4 received 8 mg/m^2^, 6 received 16 mg/m^2^, 4 received 24 mg/m^2^, 4 received 36 mg/m^2^, and 7 received 48 mg/m^2^

^b^Clinical benefit is defined as Time to Progression ≥75 days or an Objective Response (radiologic criteria)

^c^Clinical benefit rate is the number of patients with a clinical benefit ÷ number of patients in the evaluable population × 100

^d^Time to progression in days was calculated from the date of first dose of IMGN901 until the date of progressive disease

^e^Progression-free survival is defined as the time (in days) from the date of enrollment to the date of documented disease progression or death from any cause. Progressive disease was defined according to the RECIST 1.0 criteria. [New Guidelines 2000] If a patient had not progressed or died, the patient was censored on the date of the last disease assessment

**Table 4:** Patients with PR or CR by radiologic and clinical assessments

Primary tumor	IHC score^a^	Dose level (mg/m^2^)	Period	Best Response	Confirmed^b^	DOR (months)	TTP (months)	PFS (months)	OS (months)
MCC	3 + homo	36	Cycle 3	CR	Yes	69.4^c^	70^c^	75.2	75.2
SCLC	TNA	75	Cycle 1	PR	No	1.4	1.9	1.9	2.1^c^
MCC	3 + homo	60	1 month FU	Radiologic PR followed by a clinical CR	Yes	32.7^c^	34.1	34.1^c^	34.1
MCC	TNA	60	Cycle 1	PR	No	1.5	2.8	2.8	8.1

*CR* complete response, *DOR* duration of response, *PFS* progression-free survival, *IHC* immunohistochemistry, *OS* overall survival, *PR* partial response, *TNA* tissue not available, *TTP* time to progression

^a^Visual Scoring: staining intensity in test samples was scored 0–3 (0 = no staining, 1 = weak, 2 = moderate, 3 = strong) and % uniformity (focal = <25 %; heterogeneous =25–75 %; and homogeneous = >75 %)

^b^Confirmed by a repeat assessment performed prior to every other cycle (every 6 weeks) or until tumor progression

^c^Observation was censored

These findings revealed that the most notable activity was observed in patients with metastatic MCC (*n* = 23), with thirteen percent overall demonstrating responses to treatment (Table 5). One patient received a total of 6 cycles of treatment with IMGN901 at 36 mg/m^2^ and achieved a PR after cycle 1 and a CR by cycle 3. The patient remained in complete remission for nearly 6 years until dying from non-small cell lung cancer (NSCLC). Another patient had a PR after cycle 1 of treatment with IMGN901 at 60 mg/m^2^ (65 % reduction in target skin lesions), but developed reversible posterior leukoencephalopathy syndrome and was discontinued. This patient achieved a clinical CR with resolution of all skin lesions 2 years after treatment with a single course of IMGN901 and was still alive with no signs of disease progression at the last follow-up visit (~2.8 years). A third patient, who presented with widespread metastatic disease at baseline, developed PD after cycle 2 of treatment. Interestingly, whole body PET CT scan performed 8 months after study discontinuation revealed no evidence of FDG-avid disease. Subsequently, he experienced waxing and waning low-volume disease (<1 cm diameter of some lesions), but the patient was alive with no signs of PD 2.4 years after receiving the first dose of IMGN901 and without having received any additional therapy. Two patients achieved clinically relevant SD; one of them remained on study for 10 cycles, whereas the other received 21 cycles of treatment and was discontinued due to an AE of ataxia.

**Table 5:** Locally advanced and metastatic Merkel cell carcinoma patients

Age/Gender	IHC score^a^	No. cycles	Best Response	Reason for discontinuation
68/M	TNA	2	PD (cycle 2)	PD
67/F	3 + homo	1	PD (cycle 1)	PD
55/F	3 + homo	6 (36 mg/m^2^)	CR (cycle 3)	Completed study
77/M	3 + homo	1	PD (cycle 1)	PD
67/F	3 + homo	1 (60 mg/m^2^)	PR/clinical CR	AE
50/M	TNA	2	PD (cycle 2)	AE
59/M	3 + homo	4	PD (cycle 4)	PD
62/M	3 + homo	1	PD (cycle 1)	PD
55/F	3 + homo	2	PD (cycle 2)	PD
45/M	3 + homo	10 (60 mg/m^2^)	PD (cycle 8)	Withdrawal of consent
78/F	3 + homo	2	SD	AE
67/F	3 + homo	4	PD (cycle 4)	PD
62/F	3 + homo	21 (60 mg/m^2^)	SD	AE
56/M	3 + homo	2	PD (cycle 2)	PD
77/F	3 + homo	2	PD (cycle 2)	PD
68/F	2 + hetero	2	PD (cycle 2)	PD
78/M	2 + hetero	2	PD (cycle 2)	AE
75/M	TNA	2	PD (cycle 2)	PD
59/M	3 + homo	2	PD (cycle 2)	PD
67/F	TNA	2	PD (cycle 2)	PD
53/M	2 + homo	2	PD (cycle 2)	PD
54/F	3 + homo	2	PD (cycle 2)	PD
68/M	TNA	1	PR/PD (after cycle 1)	AE

*AE* adverse event, *CR* complete response, *IHC* immunohistochemistry, *PD* progressive disease, *PR* partial response, *SD* stable disease, *TNA* tissue not available

^a^Visual Scoring: staining intensity in test samples was scored 0–3 (0 = no staining, 1 = weak, 2 = moderate, 3 = strong) and % uniformity (focal = <25 %; heterogeneous =25–75 %; and homogeneous = >75 %)

Clinical activity was also observed in additional tumor types. The CBR for patients with ovarian cancer (*n* = 12) was 33.3 %. One patient in this group received 12 cycles of therapy with IMGN901 before developing PD and was alive at 1.9 years after her first dose of IMGN901. Patients with SCLC demonstrated a CBR of 21.2 % (*n* = 33), with one patient achieving an unconfirmed PR and six patients experiencing SD.

The median PFS and TTP were identical for both the overall population (2.1 months; CI = 1.2, 1.5 months) and for the subset of patients treated with 60 mg/m^2^ of IMGN901 (1.3 months; CI = 1.2, 1.6 months) (Table 2). Median OS for all patients was 9.2 months (CI = 5.7, 13.2 months), whereas median OS for patients treated at 60 mg/m^2^ was 8.1 months (CI = 5.5, 14.3 months).

### Pharmacokinetics

**Fig. 2:**
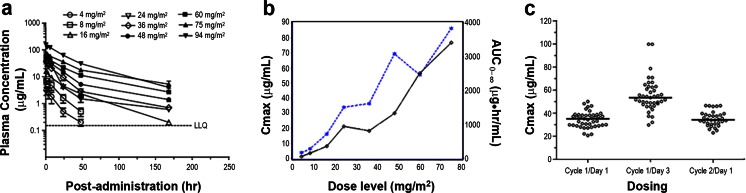
Pharmacokinetics. **a** Plasma concentration (mean ± SD) of IMGN901 over time of patients given doses at 4, 8, 16, 24, 36, 48, 60, 75, and 94 mg/m^2^ (LLQ = lower limit of quantitation). **b** C_max_ of IMGN901 (− −∗− −) and area under the concentration curve (AUC_0-∞_) (—⋄—) of patients given doses at 4, 8, 16, 24, 36, 48, 60, and 75 mg/m^2^. **c** Maximum plasma concentration (C_max_) of IMGN901 with median (**——**) for end of infusion at the first dose of cycle 1, the third dose of cycle 1, and the first dose of cycle 2 of patients given doses at 60 mg/m^2^

The maximum plasma concentration (C_max_) and area under the concentration curve (AUC_0-∞_) of IMGN901 generally increased with increasing dose (Fig. 2a, 2b). These parameters also increased with subsequent treatments within a cycle (C_max_ shown in Fig. 2c). At the 60 mg/m^2^ dose, a consistent elevation in the median C_max_ (about 1.5-fold on day 3) was observed. The half-life of IMGN901 at the 60 mg/m^2^ dose was approximately 30 to 40 h following Day 3 of cycle 1. The concentration-time profiles for total huN901 antibody were similar to those of IMGN901 (data not shown). Total huN901 antibody had a slightly longer elimination half-life than IMGN901 at doses ≥60 mg/m^2^.

## Discussion

In this study, we evaluated the safety, tolerability, pharmacokinetics and preliminary efficacy of single agent IMGN901 administered on Days 1–3 of a 21-day cycle to patients with CD56-positive solid tumors. Ninety-seven patients were accrued. Patients in the dose-escalation phase received IMGN901 at doses ranging from 4 to 94 mg/m^2^. The MTD was declared at 75 mg/m^2^ and the first seven patients enrolled into the dose-expansion phase were treated at that dose level. During drug administration, however, three patients experienced dose-limiting headache with or without meningitis-like symptoms. These events prompted the implementation of a slower infusion rate and prophylactic low-dose steroids and acetominophen, which reduced the incidence and severity of these symptoms. Similar toxicity was observed in the first-in-human study, with symptoms developing within 6 to 8 h post-infusion and resolving within 1 to 3 days [19, 23, 24]. Despite these measures, 3 of 7 patients experienced drug-related SAEs at 75 mg/m^2^, thus the RP2D was determined to be 60 mg/m^2^.

Preliminary evidence of antitumor activity of IMGN901 was noted in several CD56+ tumor types. Of the 94 patients in the efficacy evaluable population, 25.5 % experienced clinical benefit. Patients treated with 60 mg/m^2^ experienced the highest CBR (27.3 %). Four patients, three of whom were diagnosed with MCC, had objective responses. While the ORR was modest, it should be noted that all patients had previously been treated with other therapies and almost half (49 %) had received two or more prior lines of treatment.

The most interesting signals of clinical activity were seen in patients with metastatic MCC, a rare and aggressive cutaneous malignancy of neuroendocrine origin. Surgical excision of the primary tumor is the first line of treatment; however, MCC has a high propensity for local recurrence as well as regional and distant metastases [25, 26]. Current therapies have demonstrated no survival benefit for patients with distal metastatic disease [27], and for this patient population, treatment is limited to palliative care [28]. As noted above, three objective and durable responses and an overall CBR of 21.7 % were observed among the 23 patients with metastatic MCC recruited to the study. Together with preliminary signs of efficacy in SCLC and ovarian cancer patients, these results provide encouraging evidence of single-agent IMGN901 activity.

Analysis of CD56 expression demonstrated strong expression in the majority of the tumors. Despite this, not all patients derived meaningful clinical benefit. A potential explanation for this may involve acquired resistance to the microtubule-targeting payload among the heavily pre-treated patients enrolled in this study. For example, SCLC is usually responsive to initial platinum-based chemotherapy, but subsequent chemotherapy rarely results in response and shows only modest improvements in survival [29]. Furthermore, preclinical evidence suggests that induced resistance to cisplatin can render SCLC cells cross-resistant to the microtubule-disrupting vinca alkaloids [30]. Other factors that may contribute to lack of response in some patients include heterogeneous CD56 expression within a tumor, or varying rates of ADC internalization. The half-life of IMGN901 is relatively short compared with that of other humanized IgG1 antibodies; this can be explained, at least in part, by high antigen-mediated clearance due to CD56 expression on a variety of normal tissues, in particular highly abundant NK cells. This may serve to restrict IMGN901 exposure, which, in turn, may limit activity to tumors exquisitely sensitive to the maytansinoid payload.

In conclusion, IMGN901 administered IV at a dose of 60 mg/m^2^ on three consecutive days every 3 weeks exhibited acceptable safety and tolerability. Objective, durable responses in a number of patients with MCC, and durable SD observed in several previously treated patients with relapsed advanced-stage CD56+ cancers, are encouraging signs of single-agent IMGN901 activity. The clinical evaluation of IMGN901 is ongoing, with two Phase II studies, one in CD56-expressing hematological malignancies [NCT02420873] and the other in pediatric neuroendocrine-derived tumors [NCT02452554], having recently been initiated.
